# Predicting Future Service Use in Dutch Mental Healthcare: A Machine Learning Approach

**DOI:** 10.1007/s10488-021-01150-6

**Published:** 2021-08-31

**Authors:** Kasper van Mens, Sascha Kwakernaak, Richard Janssen, Wiepke Cahn, Joran Lokkerbol, Bea Tiemens

**Affiliations:** 1 Altrecht Mental Healthcare, Lange Nieuwstraat 119, 3512 PG Utrecht, The Netherlands; 2 Department of Tranzo Scientific Center for Care and Welfare, Tilburg University, Tilburg, The Netherlands; 3 Department of Health Care Governance, Erasmus University Rotterdam, Rotterdam, The Netherlands; 4 Department of Psychiatry, Rudolf Magnus Institute of Neuroscience, University Medical Center Utrecht, Utrecht, The Netherlands; 5 Trimbos Institute (Netherlands Institute of Mental Health), Utrecht, The Netherlands; 6 Behavioural Science Institute, Radboud University, Nijmegen, The Netherlands

**Keywords:** Mental healthcare, Machine learning, Resource allocation

## Abstract

A mental healthcare system in which the scarce resources are equitably and efficiently allocated, benefits from a predictive model about expected service use. The skewness in service use is a challenge for such models. In this study, we applied a machine learning approach to forecast expected service use, as a starting point for agreements between financiers and suppliers of mental healthcare. This study used administrative data from a large mental healthcare organization in the Netherlands. A training set was selected using records from 2017 (N = 10,911), and a test set was selected using records from 2018 (N = 10,201). A baseline model and three random forest models were created from different types of input data to predict (the remainder of) numeric individual treatment hours. A visual analysis was performed on the individual predictions. Patients consumed 62 h of mental healthcare on average in 2018. The model that best predicted service use had a mean error of 21 min at the insurance group level and an average absolute error of 28 h at the patient level. There was a systematic under prediction of service use for high service use patients. The application of machine learning techniques on mental healthcare data is useful for predicting expected service on group level. The results indicate that these models could support financiers and suppliers of healthcare in the planning and allocation of resources. Nevertheless, uncertainty in the prediction of high-cost patients remains a challenge.

**Supplementary Information:**

The online version contains supplementary material available at 10.1007/s10488-021-01150-6.

## Background

In high income countries, there is an estimated gap of 35–50% between demand and supply of mental healthcare resources (World Health Organization, 2013). Managing this gap is a top priority and poses a challenge to equitably allocate mental healthcare resources. An efficient mental healthcare system requires a transparent playing field in which agreements can be made between financiers and suppliers about the appropriate quantity of care. There is a time lag between the agreed budgets, the services provided and the reimbursement, which causes financial uncertainty for both parties. Therefore, there is a need for a predictive model regarding expected service use in mental healthcare (Morid et al., 2017).

Since 2008, significant changes have been implemented into the organization and financing of the Dutch mental healthcare system (Janssen, 2017). A regulated market was introduced in which insurance companies contract suppliers about the quality and quantity of care to be delivered. One of the rationales of the reform was to create transparency in expected healthcare costs by creating homogenous groups of service use. Therefore, new treatment products were introduced, called Diagnostic Related Groups (DRGs). A DRG includes a combination of diagnosis and the activities and operations performed by the care provider (Janssen & Soeters, 2010). Although patients in the Netherlands are clustered in DRGs, there still exists a large variance in service use within the groups (Boonzaaijer et al., 2015).

This variance in the use of healthcare resources shows that it is difficult to create homogenous groups or predict mental healthcare service use in general (Malehi et al., 2015). The variance is the result of a skewed distribution, in which a small group of patients is associated with a large part of the total costs (Wammes et al., 2018). In mental healthcare, this group consists of patients with complex problems in multiple areas, which have multiple care needs and a chronic course of illness (Kwakernaak et al., 2020). Because of the skewed distribution, most scientific research on predictive models use categorical outcome variables, in which healthcare resources are clustered in two or more bins, with often a focus on the ‘high-cost’ group (Boscardin et al., 2015; Chechulin et al., 2014; Colling et al., 2020; Rosella et al., 2018; Wang et al., 2017). National initiatives on predictive models, such as in the United Kingdom, Australia and New Zeeland also used a categorical outcome in which patients are assigned to clusters of service use, which can be used to adjust expected costs (Twomey et al., 2017). In the Netherlands, a similar cluster tool was developed to overcome the shortcomings of the DRG system (Working group mental healthcare severity indicator, 2015). Evaluation of the different tools concluded that the homogeneity of resources within each cluster was still suboptimal and not suited for fixed payment adjustments (Broekman & Schippers, 2017; Jacobs et al., 2019).

Creating a predictive model in which healthcare resources are defined as a categorical variable instead of a numeric outcome, is statistically convenient and better suited to deal with skewness in the data. However, there is a trade-off with the practical utility of the model. The used cut-offs in these models are often arbitrary. Moreover, the practical challenge in healthcare is methodologically simplified and information in the outcome variable is lost. For example, changes in service use within the range of a bin stay undetected, which can have serious implications in the planning and allocation of resources, especially in the high-cost categories.

In order to design a predictive model for mental healthcare resources with a numeric outcome, a possible solution lies in the large amounts of data in electronic health records that are continuously generated and stored within mental healthcare organizations (Gillan & Whelan, 2017; Shatte et al., 2019). The emerging field of machine learning allows the exploitation of large data sets and the modeling of complex underlying non-linear relationships and therefore holds potential to deal with the skewed distribution of healthcare resources (Iniesta et al., 2016).

The goal of this study is to create a machine learning prediction model for expected service use, as a starting point for agreements between financiers and mental healthcare suppliers. We aim to predict the number of treatment hours which will be reimbursed as a part of the Dutch DRG payment system. Associated with the foregoing, we aim to contribute to more equitable resource allocation and more transparency in the system.

## Methods

### Setting

This study was carried out at Altrecht Mental Healthcare, a large specialized mental healthcare organization with multiple sites in and around the city of Utrecht, The Netherlands (www.altrecht.nl). The organization offers both inpatient and outpatient facilities, and both secondary (regional) as well as tertiary (national) health services. For this study, we focus on the outpatient treatment of patients of which nearly 60% has a personality disorder, psychotic disorder or depressive disorder as main diagnosis. Treatment is financed within the National health Insurance Act (NIA).The organization provides outpatient treatment to around 13,000 patients each year with an annual budget of approximately 83 million Euros.

### Specialized Mental Healthcare Products

In specialized mental healthcare, treatments within the NIA are reimbursed via products called Diagnose Related Groups (DRGs). These contain, among other information, all activities performed within a treatment that need to be reimbursed. The price of a DRG product is always based on a treatment component containing the number of treatment hours. The duration of a DRG is up to 365 days. After 365 days, the DRG is closed and a new DRG will start if more treatment is needed. Each year, organizations in mental healthcare in the Netherlands negotiate contracts about the budgets for the next calendar year with several insurance companies, which finance care in the NIA. This study concerns an organization with six main contracts. All DRGs starting in one calendar year are part of the contract of that year.

### Data Collection

Data were collected from reimbursed DRGs starting in the years 2017 and 2018. Demographic and clinical variables were assembled and integrated with the data regarding service use (treatment hours) and organizational properties, such as duration of treatment at the organization. Data from the four most commonly used routine outcome measurement (ROM) instruments were collected as well; the brief symptom inventory and the Health of the Nation Outcome Scale for adults, elderly and children (Burns et al., 1999; Derogatis, 1983; Gowers et al., 1999; Wing et al., 1998). Only DRGs regarding regular treatment trajectories were included, which means that the so called ‘exceptional’ DRGs related to sole diagnostic examination or acute care were excluded.

### Anonymization

All data was collected and integrated within the data warehouse of the healthcare organization with a pseudonymized identifier. After the data was integrated with a SQL-script, the data was further anonymized by first removing the pseudonymized identifier such that the identifiers could not be recovered later. Next, techniques from statistical disclosure control, such as recoding and local suppression, were applied on the demographic and clinical variables to remove risk of indirect identification. Dutch law allows the use of electronic health records for research purposes under certain conditions. According to this legislation, neither obtaining informed consent from patients nor approval by a medical ethics committee is obligatory for this type of observational studies containing no directly identifiable data (Dutch Civil Law, Article 7: 458). This study has been approved according to the governance code of the Altrecht Science department.

### Input Features

The selection of variables was based on earlier attempts to develop cluster tools, literature and input from expert discussions (Kim & Park, 2019; Twomey et al., 2015). The organization treats different populations of patients within different care programs such as community-based treatments, specialized treatments or elderly mental healthcare. This results in different types of registration data available within these programs. The feature creation phase was aimed at creating comparable features applicable to all (sub) populations. Since different ROM-questionnaires were used depending on the patient’s treatment program, we used a normalized T-score, converted from the raw total ROM scores, which makes the scoring of all questionnaires comparable (Beurs et al., 2018). A T-score has a mean of 50 and a standard deviation of 10 and a score of above 55 is considered as highly severe symptoms. The T-score could be used as one feature within all four programs. In all features created, definitions were used that could be translated to other mental healthcare organizations such that the research findings are applicable to a broader spectrum. A complete list of features with a description is given in Online Appendix 1.

The features were divided into three categories: patient, supplier and service use (first 2 months). We started with a model based on the input data from the first category only. Subsequently, we created a model with both the first and second set. Lastly, we created a model with all three sets as input. The first category consisted of clinical and demographic variables. The second category was related to history of service use and characteristics of the type of treatment (measured at the start of the new DRG). The third category included features from the administrative data of appointments, meetings and other types of activities performed within the first 2 months of the DRG. The first 2 months are relative to the start date of the DRG, for example a DRG staring at the 10th of May in 2018, will contain information from the 10th of May up to the 9th of July. The time spent on these activities are part of the service use we aim to predict, so we use a part of the puzzle (the first 2 months) to predict the remaining part of the puzzle (the sum of the next 10 months). In current practice, the time lag between agreed budgets and reimbursement is about 14 months. Because of the uncertainty in budgets, negotiations and monitoring of expected costs go on continuously. Mental healthcare contracts are even negotiated ex post because they involve risks of millions of Euros in case of just one supplier. Therefore, in the third scenario, even after 2 months, it is still very relevant to reduce the uncertainty about expected costs in the upcoming 10 months. The aim of this analysis is to give insight in the trade-off between waiting for more information and apply a potentially better prediction model or apply a model directly at the start of treatment.

After deciding which variables to include into the three sets, there were some variables with missing values; *living condition (60%), education (39%), marital status (25%)* and *baseline ROM score (9%).* We imputed the label ‘unknown’ for missing values in the first three categorical variables. The numerical ROM-score was imputed with a k-nearest neighbor algorithm. All numeric variables were scaled and centered.

### Modeling

The DRG data starting in the year 2017 were used as training data. To evaluate the model, the DRGs starting in 2018 were used as test data. The training set was used to describe the population and create the models. The test set was used only once for evaluation. We built three random forest models on the three different sets of input data. A random forest is an example of ensemble learning, which is an algorithm that combines multiple predictors to make a single prediction. It has the advantage of being able to model complex interactions and non-linear relationships. The package *randomForest* as implemented in the statistical software R was used (Breiman et al., 2015; R Development Core Team, 2008). The model was trained with tenfold cross-validation with 10 repeats. The hyper parameter ‘number of trees’ was tuned on the mean absolute error with the default grid search in the *caret* package in R (Kuhn, 2008). All input variables were scaled and centralized. The prediction error is visualized by plotting the predicted number of treatment hours versus the actual number of treatment hours with the *ggplot2* package (Wickham, 2016). The importance of the variables was assessed with the *caret* package.

### Evaluation

Performance of the model was evaluated on individual and a group level, which are in this case the populations within the agreed budgets with the financier. Individual predictions were evaluated with the mean absolute error (MAE), whereas aggregated predictions on the population of each insurance company were evaluated with the mean error (ME). The 95% confidence intervals for both measures were estimated taking 1000 bootstrap samples. For comparison with other studies, we calculated R^2^ measures on the test data. We analyze the added value of the models by comparing the results to a baseline prediction model. In practice, there are six separate contracts with each of the six insurance companies within the catchment area of the organization. In the baseline model, we used the mean hours of service use within each contract per insurance company from the training data to predict the service use in each contract in the test data. The results and visual analysis on the training data are shown in Online Appendix 2.1 and 2.2.

## Results

### Demographic and Clinical Features

Slightly more than half of the patients included in the training set were female (56%) and the patients had a mean age of 44 (range 18–97). In the 75% patients for whom their marital status was registered, 26% was married. In the 40% patients for whom their living condition was registered, 40% lived alone and one in fourteen patients was either homeless, in jail or institutionalized. The demographic characteristics are shown in Table 1.

**Table 1:** Demographic description of patient population in the training data (N = 10,911)

Demographic variables	Mean	SD	%
Age	44.0	16.55	
Gender, female			55.7
Marital status			
Married			19.5
Living together, unmarried			4.8
Unmarried, never been married			
Divorced			9.5
Widowed			1.9
Unknown			25.4
Education			
High			15.6
Secondary			
Primary			1.5
Unknown			39.2
Living condition			
Single			16.2
Without partner, with children			2.4
With partner, without children			7.2
With partner, with children			7.0
Child with single parent			1.4
Child with multiple parent			3.4
Jail, institutionalized, homeless			2.8
Unknown			59.7

As shown in Table 2, the three most common diagnoses in the sample were personality disorders (22%), schizophrenia and other psychotic disorders (22%), and depressive disorders (14%). At the start of the DRG, the mean Global Assessment of Functioning (GAF) score was 49 which indicates serious symptoms or any serious impairment in social, occupational or school functioning. Of all patients, 7% started with a legal measure and 6% started their DRG with a crisis intervention, which both indicate a high urgency for care. The average T-score on baseline was 48. The duration of the start of the treatment up to the start of the DRG included in the training data was on average 5 years Table 3.

**Table 2:** Clinical description of the patient population in the training data (N = 10,911)

Clinical features	Mean	SD	%
Main diagnosis group			
Personality disorders			22.2
Schizophrenia and other psychotic disorders			21.9
Depressive disorders			13.9
Bipolar disorders			11.1
Anxiety disorders			10.4
Somatic symptom disorders			5.0
Pervasive developmental disorders			4.8
Delerium, dementia			3.6
Eating disorders			2.7
Substance related disorders			1.9
Other diagnosis			2.6
Occupational problem (DSM-IV) at start of DRG			10.9
Legal measure at start of DRG			6.9
Acute care at start of DRG			6.1
Global Assessment of Functioning at start of DRG	48.5	10.65	
T-score baseline at start of DRG	48.0	10.85	
Treatment duration from start DRG, years	4.6	6.06

**Table 3:** Results on test data (2018, N = 10,201)

	N	Mean hours	Baseline model (R^2^ = 0.00)				Model1 (R^2^ = 0.18)			
			ME	CI	MAE	CI	ME	CI	MAE	CI
1	3860	60.27	− 1.62	− 3.66–0.44	45.86	44.42–47.36	0.51	− 1.38–2.42	40.09	38.8–41.47
2	1355	63.38	− 1.64	− 5.3–1.89	49.7	47.29–52.25	3.25	0.02–6.55	43.19	40.89–45.5
3	300	68.54	7.58	− 1.99–16.87	57.18	50.74–63.59	9.95	1.83–18.12	51.32	45.36–57.13
4	1472	62.77	− 2.02	− 5.27–1.37	47.08	44.63–49.45	3.89	0.91–6.94	41.14	38.9–43.22
5	1431	59.25	1.70	− 1.55–4.84	44.5	42.26–46.93	6.84	3.85–9.74	40.51	38.28–42.71
6	1783	63.68	0.99	− 2.08–4.13	49.06	46.92–51.21	4.12	1.23–7.07	43.56	41.6–45.62
Total	10,201	61.74	− 0.49	− 1.81–0.78	47.25	46.35–48.14	3.16	1.98–4.32	41.64	40.81–42.45

	N	Mean hours	Model2 (R^2^ = 0.28)				Model3 (R^2^ = 0.54)			
			ME	CI	MAE	CI	ME	CI	MAE	CI
1	3860	60.27	− 0.01	− 1.72–1.78	35.86	34.58–37.21	− 0.18	− 1.55–1.14	27.36	26.31–28.44
2	1355	63.38	2.17	− 0.84–5.27	39.08	36.85–41.28	0.86	− 1.56–3.35	29.84	28.07–31.69
3	300	68.54	6.19	− 1.05–13.18	42.44	37.3–47.6	− 0.48	− 6.46–5.62	32.37	27.58–37.18
4	1472	62.77	2.03	− 0.88–4.75	36.86	34.76–39.05	0.36	− 1.96–2.62	28.54	26.7–30.35
5	1431	59.25	4.66	1.90–7.34	35.23	33.15–37.2	1.26	− 0.92–3.37	26.55	24.88–28.28
6	1783	63.68	3.31	0.60 – 6.00	39.77	37.82–41.77	0.52	− 1.62–2.6	30.12	28.53–31.77
Total	10,201	61.74	1.99	0.87–3.02	37.21	36.42–38	0.35	− 0.52–1.26	28.37	27.72–29.05

Aggregated predictions on test data for each insurance company population

*ME* mean error, *MAE* mean absolute error, with 95% bootstrapped confidence intervals

### Performance of the Machine Learning Model on Test Data

The output of the baseline model and the three machine learning models on the test data is shown in Table 3. The six rows resemble the six contracts with each insurance company, with the number of patients within the contract (N) and the actual mean hours. For each model, the mean error (group level) and mean absolute error (patient level) were estimated. There was considerable improvement in model2 over model1 and model3 over model2. Compared to the baseline model, all three models improved performance at the individual level. Only model3 showed considerable improvement at the group level.

In the total population of 10,201 patients, the actual hours of mental healthcare averaged 62. Model3 resulted in an average error of 0.35 h (21 min) at the group level, which is 0.5% of the mean, and an average absolute error of 28 h at the patient level, which is 45% of the mean.

### Individual Predictions Compared to Actual Hours

**Fig. 1:**
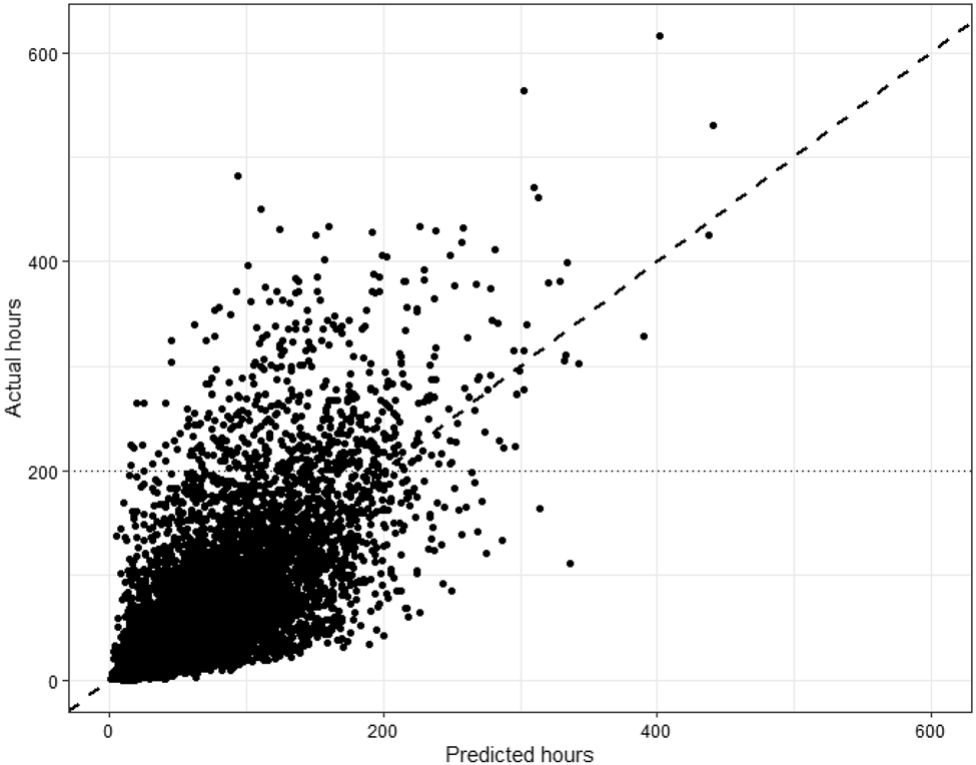
Scatterplot of predicted versus actual hours of model3 on test data 2018 (N = 10,201)

A visual analysis of the prediction on the test data is shown in the scatterplot in Fig. 1. We observe both under- and overestimation by the distance of dots to the dashed diagonal line. There is a clear case of skewness, a high-cost group of 5% of the DRG products (> 200 h on the y-axis), which contain 22% of the total hours. Furthermore, we observe a cloud of dots above the diagonal line within this group, which indicate substantial and systematic underestimation of the actual hours.

### Variable Importance

The top five most predictive variables are shown in Table 4. The most important patient variables (model1) included functioning and the severity of clinical symptoms, expressed with a GAF-score or measured with a ROM-measurement. The most important organizational variables (model2) were related to previous healthcare use and duration of treatment. In model3, the most important variables relate to the total time as well as the duration of hours spent on appointments and activities in the first 2 months of treatment.

**Table 4:** Top five most predictive variables for each model with scaled (relative) variable importance values

Rank	Model1		Model2		Model3	
1	GAF	100	Hours previous year	100	Time spent in hours in month 2	100
2	T-score baseline (ROM)	76	Duration of treatment at start of DRG	50	Time spent in hours in month 1	26
3	Age	70	Crisis situation previous year	44	Duration of treatment at start of DRG	22
4	Raw score baseline (ROM)	57	T-score baseline (ROM)	38	Time spent on intake activities in month 1 and/or 2	20
5	Legal measures	54	Age	37	Time spent on treatment appointments in month 1 and/or 2	20

The variable that contributed the most to model performance is set to 100 and the contribution of the other variables are related to the most contributing variable

## Discussion

This is one of the few studies that use machine learning on a large database to predict a numeric outcome on mental healthcare service use. The goal of this study is to create a random forest prediction model for expected service use, as a starting point for contracting processes between financiers and mental healthcare suppliers in the Netherlands. A random forest algorithm was used on a large electronic health record database to predict the number of hours in the DRGs of a large mental healthcare organization in the Netherlands.

Three models were created to predict the quantity of service use. The first model, using only patient-related data resulted in a group level error of 5% and an absolute (patient level) error of 67% of the mean. The second model, adding organizational data and data about past service use, reduced the error to 3% and the absolute error to 60% of the mean. The third model, adding data related to the first 2 months of the DRG, further reduced error to 1% and the absolute error to 46% of the mean.

We found that comparing the results to other studies is difficult because only a few studies used a numeric outcome with a train-test or other out of sample designs. With those studies, a direct comparison of the mean absolute error is not valid because the error depends on the distribution of the outcome in the dataset. Moreover, the type of input data available was not the same. As an indication, the study of Kuo et al. (2011) predicted costs and reported a R^2^ of 0.48 and a mean average error of $507, which was 75% of the mean. In a study of Bertsimas et al. (2008), an absolute error of €1,977 was found, which was 79% of the mean. The absolute error in our three models ranges from 46 to 67% of the mean. Another comparison can be made within the Dutch context. The evaluation of the Dutch cluster tool reported an R^2^ of 0.06, but no train-test design was used, which could result in an overestimation of the performance of the model. In line with Yarkoni (2017), we argue that future studies and national initiatives about predicting service use should use fundamental concepts of machine learning and focus on making generalizable predictions. Nonetheless, compared to the cluster tool, which only used patient-related data, model1 already showed a R^2^ of 0.18 on the test data, which implies that the model has higher predictive value.

We determine the practical implication of our model by translating the statistical performance to the case of our study, in which the healthcare organization had to establish six financial agreements in 2018. The models developed on the training data (2017) are used to predict the six budgets. The error of the best model is translated to a financial risk in Euros and is compared to risk of the baseline model. The financial risk is calculated by taking the absolute sum of the errors in each contract and multiply it with 110 Euros per hour, which is the hourly reimbursement value. The error in each contract is defined as the mean error times the number of patients. In our example, there would be a total error of 17,190 h in the baseline model, valued at €1,970,100. When using model3 to predict the budgets, there would be a total error of 5266 h, valued at €579,228. The error is reduced by 71%, a reduction of financial risk for the organization of €1.4 million on a budget of €83 million.

The skewness in the healthcare data remains a challenge. From the visual analysis in Fig. 1 we observed that there is a clear presence of a high-cost group and that we systematically under predict this group, which means that it is hard to distinguish this group from other patients in advance. In line with results from Yang et al. (2018), we expected that past year service use could be used within a machine learning model to predict this group. However, random forest is not immune to the challenge of skewness. Moreover, Johnson et al. (2015) found that high-care service use can be temporary and instable at the individual patient level. This proposes a challenge in practice, because small changes in the prevalence of this group can have a high impact on agreed budgets between financers and suppliers (Eijkenaar & van Vliet, 2017).

An important finding in the variable importance was that ROM-measurements appeared more important predictors than predictors capturing DSM-IV criteria. Therefore, we should look beyond the DSM-IV criteria when creating predictive models. The data in model2 substantially improved performance over model1, which means that predictive tools should also aim to incorporate features about past service use, such as volume or the presence of acute care in the near past. This is in line with another Dutch study in which past service use has been proven to improve predictive performance (van Veen et al., 2015). Data from model3 improved performance, which means that using information from the first 2 months of treatment is valuable in predicting service use for the remaining duration of the DRG.

## Limitations

The most important limitation is that data from only one organization was used. In order to further analyze the implication on national healthcare policies, a multisite research should be conducted. Second, our analyses are based on real-world registration data, which are limited in data quality. Furthermore, we did not have access to all data in the EHR and were dependent on the available data that could be automatically extracted from the data warehouse. Therefore, potentially predictive information such as medication use could not be used as input features. In this study we only applied a random forest algorithm and did not compare the results to other machine learning algorithms. A random forest is relatively simple and flexible, such that the method can be easily replicated by other researchers. However, a more complex algorithm could potentially improve predictive accuracy.

## Strengths

The major strength of this paper is that we used a machine learning approach on a large available dataset from a mental healthcare organization. We chose to predict the number of hours instead of the price in Euros to make the model more applicable to other types of financing systems based on treatment sessions or hours. As to our knowledge, this is one of the few articles using a machine learning approach, with a train-test design, to predict a skewed numeric outcome. Predictions could be further improved with data from other institutions, such as insurance claim data. Furthermore, implementing such machine learning models in mental healthcare contributes to transparency of service use and reduces uncertainty in financial risk for healthcare financiers and suppliers.

## Conclusion

The application of machine learning techniques on mental healthcare data might be useful to forecast expected service use on the group level. The results indicate that these models support healthcare organizations and financiers to reach agreements on annual budgets. Broader multisite research is needed to develop a national model. Nevertheless, uncertainty in the prediction of high-cost patients remains a challenge in the allocation of resources.

## Supplementary Information

Below is the link to the electronic supplementary material.

Supplementary file1 (DOCX 42 kb)
