# Supplementary material for: Predicting Future Service Use in Dutch Mental Healthcare: A Machine Learning Approach
Source: Adm Policy Ment Health. 2021 Aug 31;49(1):116–24. doi: 10.1007/s10488-021-01150-6 (PMC8732820; doi:10.1007/s10488-021-01150-6)
Supplement: Supplementary file 1 — Supplementary file1 (DOCX 42 kb) [file 10488_2021_1150_MOESM1_ESM.docx]

**Appendix 1.** Feature list

| Feature | Model | Variable type | Description |
| --- | --- | --- | --- |
| Hours of treatment in DRG | outcome | numeric | Total hours reimbursed in DRG product |
| Number of legal measures | 1 | numeric | Number of legal measures during start of DRG |
| Legal measure (type = detention) | 1 | dichotomous | Yes/no legal measure at start is detention |
| Legal measure (type = Court authorization) | 1 | dichotomous | Yes/no legal measure at start is court authorization |
| Legal measure (type = other) | 1 | dichotomous | Yes/no legal measure at start is of type other |
| Crisis situation | 1 | dichotomous | Patient started regular treatment after having been in crisis situation |
| Age | 1 | continous | Age of patient at start of DRG |
| Gender | 1 | dichotomous | Gender of patient |
| Marital status | 1 | categorical | Marital status (without history) |
| Education | 1 | categorical | Level of education (without history) |
| Living condition | 1 | categorical | Living condition (without history) |
| Main diagnosis group | 1 | categorical | Main diagnosis group at start of DRG |
| Social economic status | 1 | numeric | Variable created from postal code with a table from Statistics Netherlands |
| Global Assessment of Functioning | 1 | numeric | GAF score at start of DRG |
| Comorbidity Substance abuse | 1 | dichotomous | Yes/no comorbidity at start of DRG |
| Comorbidity Anxiety disordser | 1 | dichotomous | Yes/no comorbidity at start of DRG |
| Comorbidity Bipolar disorder | 1 | dichotomous | Yes/no comorbidity at start of DRG |
| Comorbidity Dementia | 1 | dichotomous | Yes/no comorbidity at start of DRG |
| Comorbidity Depression | 1 | dichotomous | Yes/no comorbidity at start of DRG |
| Comorbidity Eating disorder | 1 | dichotomous | Yes/no comorbidity at start of DRG |
| Comorbidity Personality disorder | 1 | dichotomous | Yes/no comorbidity at start of DRG |
| Comorbidity Pervasive | 1 | dichotomous | Yes/no comorbidity at start of DRG |
| Comorbidity Somatoform disorders | 1 | dichotomous | Yes/no comorbidity at start of DRG |
| Comorbidity Schizofrenia | 1 | dichotomous | Yes/no comorbidity at start of DRG |
| Comorbidity Other diagnosis | 1 | dichotomous | Yes/no comorbidity at start of DRG |
| Social dysfunctioning | 1 | dichotomous | Yes/no at start of DRGaxis IV DSM-IV |
| Occupational problems | 1 | dichotomous | Yes/no at start of DRGaxis IV DSM-IV |
| Other additional problems | 1 | dichotomous | Yes/no at start of DRGaxis IV DSM-IV |
| Financial problems | 1 | dichotomous | Yes/no at start of DRGaxis IV DSM-IV |
| Raw score baseline (ROM) | 1 | numeric | Raw total score on ROM measurement at start of DRG (BSI, HoNOS children, adult and senior) |
| T-score baseline (ROM | 1 | numeric | Converted T-score on ROM measurement at start of DRG |
| Serial number of treatment | 2 | numeric | The number of which consecutive treatment the DRG belongs to |
| First year of treatment | 2 | dichotomous | Yes/no whether DRG relates to first year of treatment |
| Care program | 2 | categorical | To which care program was patient referred to at start DRG |
| Referal type | 2 | categorical | Type of referral (primary, secondary, police, other) |
| Duration of treatment at start of DRG | 2 | numeric | Number of years from start treatment at organization and start DRG |
| Past year hospitalization | 2 | dichotomous | Was the patient hospitalized at the organization in the past year |
| Past year total hours of treatment | 2 | numeric | Total number of hours spent on patient in last year |
| Past year use of emergency service | 2 | dichotomous | Did the patient receive care from emergency service in the organization in the last year |
| Time spent in hours in month2 | 3 | numeric | Total hours spent in month 2 of the DRG |
| Time spent in hours in month1 | 3 | numeric | Total hours spent in mont 1 of the DRG |
| Time spent on meetings in month 1 and 2 | 3 | numeric | Total hours spent in month 1 and 2 on activities of type 'meeting' |
| Time spent on administrative activities month 1 and 2 | 3 | numeric | Total hours spent in month 1 and 2 on activities of type 'administrative' |
| Time spent on coordination of care in month 1 and 2 | 3 | numeric | Total hours spent in month 1 and 2 on activities of type 'coordination of care' |
| Time spent on the intake activities in month 1 and 2 | 3 | numeric | Total hours spent in month 1 and 2 on activities of type 'intake' |
| Time spent on treatment activities in month 1 and 2 | 3 | numeric | Total hours spent in month 1 and 2 on activities of type 'meeting' |
| Time spent by a psychologist in month 1 and 2 | 3 | numeric | Total hours spent in month 1 and 2 by a profesion of type 'psychologist' |
| Time spent by a psychiatrist in month 1 and 2 | 3 | numeric | Total hours spent in month 1 and 2 by a profesion of type 'psychiatrist' |
| UrenOverigDirect | 3 | numeric | Total hours spent in month 1 and 2 on activities of type 'other' |
| Hospitalization in month 1 | 3 | dichotomous | Yes/no was patient hospitalized during first month of DRG |

**Appendix 2.1.** Non cross-validated performance of machine learning models on training data from 2017^a^

|  |  |  | Model1 (R^2^ = 0.81) | | | | Model2 (R^2^ = 0.86) | | | | Model3 (R^2^ = 0.92) | | | |
| --- | --- | --- | --- | --- | --- | --- | --- | --- | --- | --- | --- | --- | --- | --- |
|  | N | Mean hours | ME | CI | MAE | CI | ME | CI | MAE | CI | ME | CI | MAE | CI |
| 1 | 5371 | 58.65 | .59 | -.14 - 1.36 | 18.78 | 18.20 - 19.35 | .52 | -.12 - 1.19 | 14.82 | 14.33 - 15.33 | .64 | .17 –  1.11 | 10.73 | 10.33 - 11.1 |
| 2 | 1404 | 61.75 | 1.41 | -.13 - 2.84 | 19.65 | 18.62 - 20.73 | .77 | -.54 - 2.10 | 16.49 | 15.48 - 17.49 | .12 | -.89 - 1.15 | 11.96 | 11.15 - 12.72 |
| 3 | 200 | 76.12 | 1.28 | -3.64 - 6.47 | 26.14 | 22.24 - 29.94 | -.37 | -4.80 - 4.01 | 21.2 | 17.9 - 24.45 | -.86 | -4.00 - 2.27 | 13.6 | 11.04 - 16.24 |
| 4 | 1398 | 60.75 | 2.13 | .7 - 3.57 | 18.99 | 17.94 - 20.04 | 1.37 | .17 –  2.55 | 15.26 | 14.3 - 16.22 | 1.14 | .26 –  2.02 | 10.71 | 10.01 - 11.42 |
| 5 | 709 | 60.94 | 2.06 | .05 - 3.96 | 18.96 | 17.54 - 20.4 | 1.68 | .06 –  3.38 | 15.61 | 14.37 - 16.9 | 1.13 | -.12 - 2.42 | 11.73 | 10.7 - 12.74 |
| 6 | 1829 | 64.68 | 1.34 | -.08 - 2.85 | 20.69 | 19.64 - 21.74 | .39 | -.86 - 1.69 | 17.05 | 16 - 18.03 | .44 | -.48 - 1.39 | 12.12 | 11.41 - 12.83 |
| Total | 10911 | 60.80 | 1.13 | .58 - 1.67 | 19.38 | 19.00 - 19.79 | .70 | .24 –  1.17 | 15.63 | 15.28 – 16.00 | .61 | .26 –  .95 | 11.23 | 10.96 - 11.51 |

^a^Aggregated predictions on training data for each insurance company population. ME = Mean Error, MAE = Mean Absolute Error, with 95% bootstrapped confidence intervals.

**Appendix 2.2.** Scatterplot of predicted versus actual hours of model3 on training data from 2017**
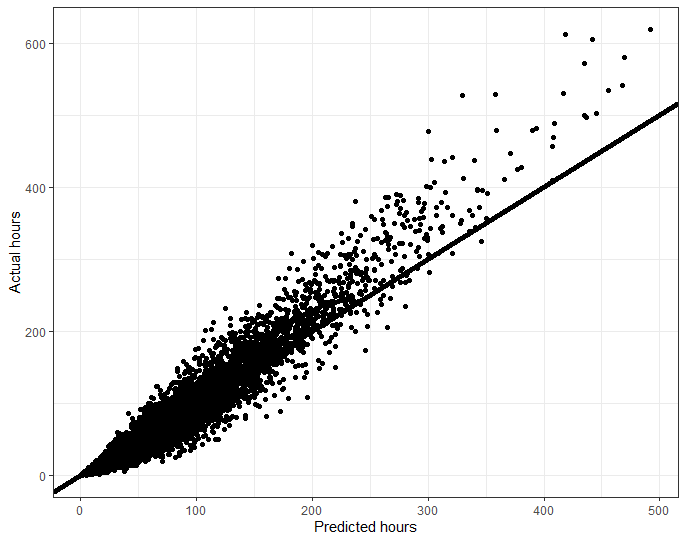
**
